# Early Lyme neuroborreliosis manifesting as brachial plexopathy and meningitis in Northwestern Ontario, Canada: A case report

**DOI:** 10.1097/MD.0000000000031576

**Published:** 2022-11-11

**Authors:** Kaien Gu, Carl Boodman, Pamela Orr, Terence Wuerz

**Affiliations:** a Department of Internal Medicine, Max Rady College of Medicine, University of Manitoba, Canada; b Departments of Community Health Sciences and Medical Microbiology, Max Rady College of Medicine, University of Manitoba, Canada.

**Keywords:** case report, Lyme disease, Lyme neuroborreliosis

## Abstract

Lyme disease is a tick-borne disease caused by the spirochete *B. burgdorferi*, and patients often present with symptoms comparable to a viral-like illness. The diagnosis can be challenging given its wide range of manifestations and diagnostic testing can take days or longer. Here, we present a case of Lyme disease presenting as brachial plexopathy and meningitis.

**Patient concerns:**

A 76-years-old male presented to a tertiary-care hospital with left arm weakness and neck pain.

**Diagnosis:**

Our patient was diagnosed with Lyme neuroborreliosis and had positive serology, including enzyme immunoassay and Western blot.

**Interventions:**

Our patient received 17 days of ceftriaxone (2g IV daily) followed by oral doxycycline (100mg bid).

**Outcomes:**

Over the subsequent year, our patient had eventual complete recovery in muscle strength and sensation, with slower improvement to the cervical neck and left arm pain.

**Lessons:**

Incidence of Lyme disease is increasing in North America, and the disease has a wide range of symptoms. Lyme neuroborreliosis (LNB) is 1 presentation and can present with early or late manifestations; clinicians should maintain a high index of suspicion and begin empiric treatment in individuals with a clinical syndrome consistent with LNB. Early LNB manifestations have onset within 6 months of infection and include cranial and peripheral neuropathy, radiculitis, and aseptic meningitis; late LNB encompasses a chronic encephalomyelitis.

## 1. Introduction

Lyme disease is a tick-borne disease caused by the spirochete *B. burgdorferi*, and patients often present with symptoms comparable to an influenza-like illness. Over the past decades, the incidence of Lyme disease in North America has been increasing. We present a case of Lyme disease presenting as brachial plexopathy and meningitis. Our case raises awareness of the varied clinical manifestations of Lyme neuroborreliosis (LNB).

## 2. Case report

In August 2019, 33 days before admission to our hospital, a 76-years-old man with a previous history of dyslipidemia, hemochromatosis, and benign prostatic hypertrophy presented to the emergency department of a community hospital in Northwestern Ontario. He provided written consent for the publication of this case report. He had spent much of the preceding late spring and summer months out-of-doors in Northwestern Ontario. He complained of a 3-days history of “body aches” and headache. He denied fever, arthralgia, rash, nausea, or vomiting. He gave a history of noticing what he thought was a mosquito bite on his anterior right upper chest, which became increasingly red and swollen. He did not see any ticks attached to his skin and did not describe seeing the “central clearing” indicative of a “target lesion.” On examination, he was afebrile, and his blood pressure and heart rate were normal. He had an area of erythema, warmth, and tenderness surrounding a central punctum in the anterior right upper chest without fluctuance, pus, or central clearing suggestive of a target lesion. A diagnosis of purulent cellulitis was made, and he was prescribed a 7-days course of cephalexin. His symptoms resolved.

He returned to the same emergency department 24 days later with a 4-days history of mild headache and bilateral, paroxysmal “shock-like” pains radiating from his shoulders to his arms and chest. Strength and range-of-motion testing of the shoulders were unremarkable, but flexion and abduction at the shoulder elicited pain radiating down his arms to the hands. His neck was supple. There were no skin lesions or lymphadenopathy. Examinations of the chest, heart, and abdomen were normal. Electrocardiogram, cardiac enzymes, and chest x-ray were normal. Two-tier Lyme serology with an enzyme immunoassay and, if positive, a Western blot was ordered. A tentative diagnosis of viral syndrome was made. The patient was advised to take ibuprofen as required and follow-up with his family doctor.

Four days later, the patient experienced new left arm and hand weakness, prompting him to present to the emergency department of a hospital in Winnipeg, Manitoba. Neurologic examination revealed weakness of the left upper extremity. A CT exam of the brain and Circle of Willis with angiography was unremarkable. The emergency department physician diagnosed a left C6-7 nerve route lesion, ordered an urgent outpatient uninfused MRI (results described below), and recommended urgent follow-up with our patient’s family physician.

Five days later, the patient presented to another tertiary care hospital in Winnipeg with increasing left arm weakness and neck pain. He endorsed occasional night sweats without fevers or rigors. On examination, he was afebrile, and vital signs were unremarkable. His cardiac and dermatologic examinations were normal. His neck was flexed forward, and he had swelling, tenderness, and mild erythema to the anterior left shoulder, with marked weakness throughout his left upper extremity and decreased pinprick sensation. Deep tendon reflexes to both upper extremities were absent, but normal to the lower extremities. Strength and sensation of the lower extremities were normal, as were examinations of the cranial nerves, speech, and higher cognitive functions.

Complete blood count and differential, immunoglobulins, complements, anti-neutrophil cytoplasmic antibodies, syphilis serological testing, and an electrocardiogram were normal. The recent spine MRI 4 days prior had revealed multilevel disc degenerative changes and degenerative changes to facet joints, without atrophy or pathologic signal changes to the spinal cord. A repeat MRI of the spine and left brachial plexus now showed increased signal uptake along the ventral aspect of the spinal cord at T3 and along the left brachial plexus, which was felt to be in keeping with polyradiculitis. A lumbar puncture showed markedly elevated protein of 2.11 grams/liter (normal range 0.2‐0.4 g/L), with a cerebrospinal fluid (CSF): serum glucose ratio of 0.5. Microscopic analysis of the CSF revealed a nucleated cell count of 273 cells per high powered field (normal 0‐5), with 90% lymphocytes and no red blood cells. CSF cultures were negative for bacterial or fungal growth. The patient was admitted and empirically started on acyclovir, ampicillin, and doxycycline; differential diagnosis for his suspected aseptic meningitis included LNB, as well as *Listeria monocytogenes* or viral causes. The following day, the serology results taken in the emergency department of the Northwestern Ontario hospital became available and were communicated to his Winnipeg doctors. Lyme serology was positive with a screening enzyme immunoassay and a positive Western blot IgM, but negative for IgG. CSF polymerase chain reaction for *Borrelia* species was negative. Paired Lyme serology testing from CSF & serum was not performed.

The patient was diagnosed with meningoradiculitis as a manifestation of early LNB. Previous antimicrobials were discontinued in favor of ceftriaxone 2g IV daily. A transthoracic echocardiogram was subsequently performed and demonstrated mild left ventricular diastolic dysfunction. He was discharged approximately 2 weeks later and completed 17 days of ceftriaxone, followed by 2 weeks of oral doxycycline 100mg PO bid. Over the subsequent year, he had eventual complete recovery in muscle strength and sensation, with slower improvement to the cervical neck and left arm pain.

## 3. Discussion

**Figure 1.**
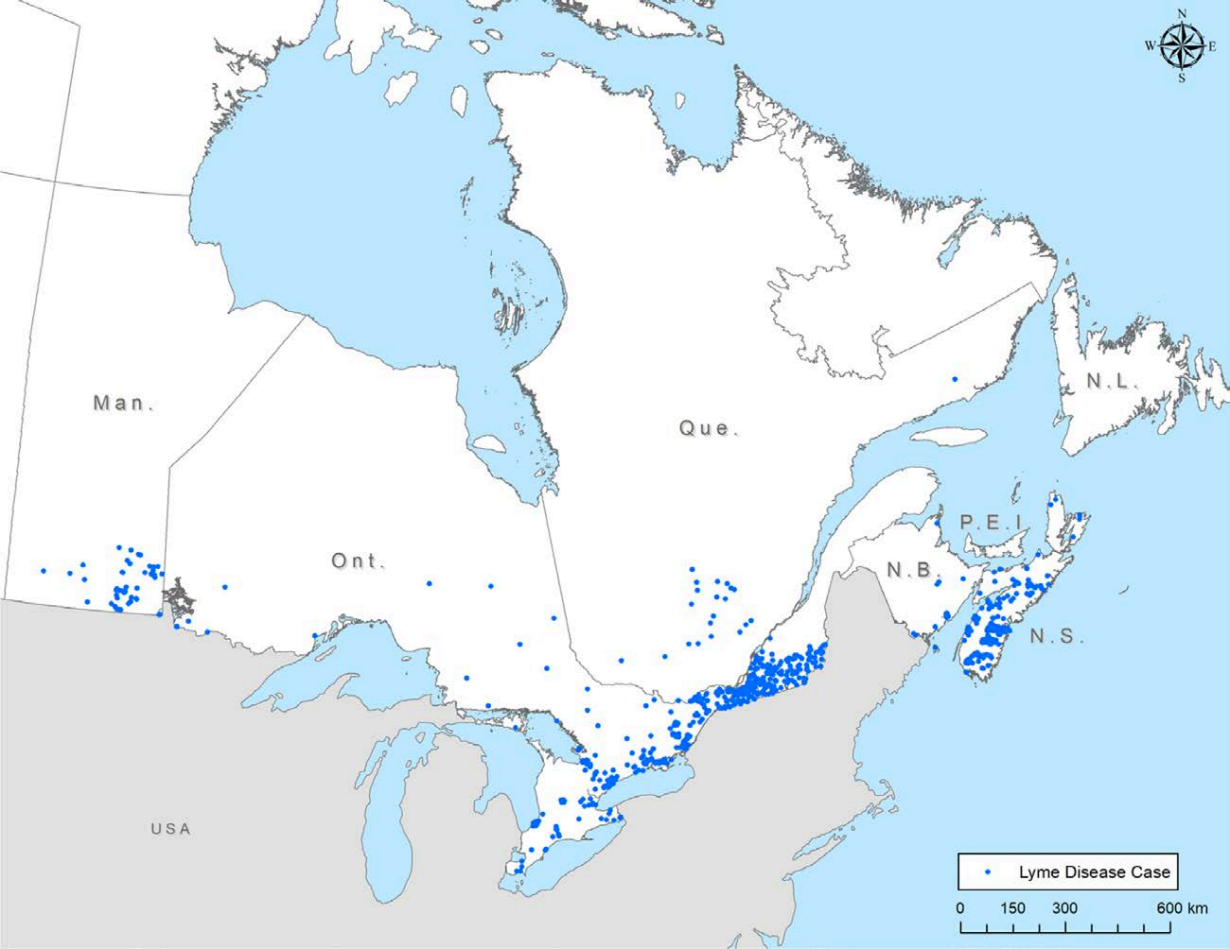
Reported locations of Lyme disease acquisition in Canada, 2018.^[10]^

Lyme disease is caused by the spirochete *B. burgdorferi*. In North America, it is transmitted by ticks of the *Ixodes* genus, or blacklegged ticks. Incidence is highest in the Northeastern United States and around the Great Lakes regions of both Canada (Fig. 1) and the United States.^[1]^ Newly endemic areas are found annually and further changes are expected in coming years with the emergence of new pathological *Borrelia* strains, such as *B. mayonii*.^[2]^

The earliest symptoms of Lyme are usually nonspecific, with fevers, myalgias, and fatigue masquerading as a “flu-like illness” 3 to 30 days following infection. In up to 80% of cases, symptoms are accompanied by an expansile, annular rash of erythema migrans (EM). The early symptoms and EM rash are self-limited and generally resolve within 1 month.

LNB refers to a range of neurologic abnormalities associated with *B. burgdorferi* infection, and are described, broadly, as early or late LNB based on whether symptoms have been occurring for more than 6 months.^[3]^ Neurological manifestations of early LNB often occurs a few weeks, though as early as 5 days, after infection and may occur after EM has faded. Cranial neuropathies, particularly of the facial nerve, are the most common manifestation of early LNB, and have been reported in 15% of patients with untreated EM.^[4]^ Other relatively common manifestations of early LNB include painful radiculoneuritis or plexopathies, aseptic meningitis, and rarely, mononeuritis multiplex or encephalomyelitis. Meningo(poly)radiculitis, or Bannwarth syndrome, consists of the “triad” of painful radiculoneuritis, lymphocytic pleocytosis in the CSF, and cranial neuropathy.^[5]^ This syndrome has been described more frequently in Europe, commonly in association with *Borrelia garinii* infection, a causative agent of LNB endemic to the region. Late LNB has been less commonly described compared to early neurologic manifestations of the disease, with presentations including polyneuropathy and a slowly progressive encephalomyelitis.

The early recognition of Lyme is important for appropriate management; however, the paucity of classic features can present a challenge. Most patients do not recall a tick bite, and EM can also go unnoticed.^[6]^ Further, unless diagnostic tests for Lyme, such as serology, are requested, few clues on standard laboratory or radiographic evaluation would otherwise alert a clinician to the possibility of LNB. As a result, LNB is often misdiagnosed or underdiagnosed, with significant social and financial costs.^[7]^ Key factors that should raise the suspicion of LNB include time of year and geographical location.

The diagnosis of LNB rests on objective evidence of a compatible clinical syndrome, potential epidemiologic exposure, and positive laboratory evaluation for Lyme. Serum antibody testing, using approved 2-tier *Borrelia* serology, is highly reliable for evidence of exposure to *Borrelia burgdorferi*; important caveats include initially false-negative evaluation (when measured within 4‐6 weeks of infection) and that results may take days to return.^[8]^ Public Health Ontario utilizes 2-tier Lyme serology testing with an initial *Borrelia* VlsE1/pepC10 IgM/IgG enzyme-linked immunosorbent assay; all reactive or indeterminate samples undergo subsequent *B. burgdorferi* IgM and IgG Western Blot. Simultaneous testing of the serum and CSF, to determine the CSF: serum antibody index, is also recommended in LNB involving the central nervous system. Polymerase chain reaction amplification of *B. burgdorferi* DNA from CSF samples may be attempted; however, due to the paucibacillary nature of the disease within the CSF, the sensitivity of this test is low.^[3,9]^

The prognosis for patients with promptly treated early LNB is generally favorable, with near complete or complete recovery of neurologic function. The mainstay of treatment for early LNB is a 14‐21-days course of an appropriate, Lyme-specific antimicrobial agent including IV ceftriaxone or penicillin G, as well as oral doxycycline. For patients with central nervous system (CNS) involvement, current guidelines recommend using the IV route for initial therapy.^[9]^ For patients suspected of having early LNB patients with CNS involvement, empiric therapy should be offered.

## 4. Conclusion

Lyme disease, caused by *Borrelia burgdorferi*, is increasing in North American. Early symptoms are protean. A history of EM rash or black-legged tick bite may go unrecognized. Therefore, patients with new cranial (especially facial) neuropathy, painful radiculitis, or aseptic meningitis, who present in a Lyme- endemic area during or shortly after tick season should be alert to the possibility of early LNB. Two-tiered Lyme serology is a sensitive method for diagnosis but should be repeated if initially negative results are received and the potential Lyme exposure occurred < 6 weeks prior. For individuals with suspected LNB and CNS involvement, clinicians are urged to consider empiric therapy with IV ceftriaxone or penicillin G, while undertaking urgent evaluation.

## Author contributions

**Conceptualization:** Kaien Gu, Terence Wuerz.

**Writing – original draft:** Kaien Gu, Terence Wuerz.

**Writing – review & editing:** Kaien Gu, Carl Boodman, Pamela Orr, Terence Wuerz.
